# Positive *Cutibacterium acnes* Intervertebral Discs Are Not Associated with Subsidence Following Anterior Cervical Discectomy and Fusion at 3 or 6 Months

**DOI:** 10.3390/jcm13185619

**Published:** 2024-09-22

**Authors:** Jael Camacho, Jake Carbone, Rohan I. Suresh, Shivam Khanna, Ivan B. Ye, Alexandra E. Thomson, Jacob Bruckner, Rohan Gopinath, Shane McGowan, Nathan O’Hara, Louis J. Bivona, Julio J. Jauregui, Daniel L. Cavanaugh, Eugene Y. Koh, Steven C. Ludwig

**Affiliations:** Division of Spine Surgery, Department of Orthopaedics, University of Maryland School of Medicine, Baltimore, MD 21201, USAjjauregui@som.umaryland.edu (J.J.J.);

**Keywords:** *Cutibacterium acnes*, anterior cervical discectomy and fusion (ACDF), degenerative disc disease (DDD), subsidence

## Abstract

**Background/Objectives:** *Cutibacterium acnes* (*C. acnes*), formerly known as *Propionibacterium acnes* (*P. acnes*), is an anaerobic, low-virulent bacterium that has been associated with postoperative infections of the shoulder, knee, and cervical spine. Recent studies have highlighted an association between *C. acnes* and the development of degenerative disc disease (DDD). The aim of this study is to ascertain whether *C. acnes* increases the risk of subsidence following anterior cervical discectomy and fusion (ACDF). **Methods:** After IRB approval, consecutive patients undergoing elective ACDF for DDD from 2017 to 2018 were enrolled in this prospective cohort study. Intervertebral disc samples were taken at each affected level and cultured. A total of 66 patients with radiographic follow-ups were included in the final analysis. The extent of subsidence and cervical lordosis was determined immediately postoperatively and at the 3- and 6-month follow-ups. **Results:** No significant difference in subsidence was observed at 3 months (*p* = 0.07) or 6 months (*p* = 0.11) between culture-positive and -negative cohorts. Additionally, there was no significant difference detected in the change in cervical lordosis observed at 3 months (*p* = 0.16) or 6 months (*p* = 0.27) between culture-positive and -negative cohorts. For the most inferiorly fused segment, there was a significant difference in subsidence observed at 3 months (1.5 mm, 95% CI: 0.2–2.7 mm, *p* = 0.02) but not at 6 months (*p* = 0.17). **Conclusions:** Intervertebral discs with a positive *C. acnes* culture were not associated with greater levels of subsidence at 3 or 6 months following ACDF for DDD. Further research is necessary to endorse these results and to gauge the clinical significance of *C. acnes* infection.

## 1. Introduction

*Cutibacterium acnes* (*C. acnes*), formerly known as Propionibacterium acnes (*P*. acnes), is a low-virulence, anaerobic, and Gram-positive rod often seen in sebaceous glands [1]. *C. acnes* is predominantly seen in the sebaceous glands of the face and neck, but the topographical presence of the bacterium is dependent on multiple factors and can be highly personalized to the individual [1]. Variables such as body temperature, pH, moisture, and sebaceous follicle density can all affect the presence of *C. acnes* on one’s skin [1]. *C. acnes* is one of the predominant organisms that constitute our skin flora and, due to its preference for sebaceous sites, it is currently being seen that this bacteria can infect other parts of the body and cause numerous inflammatory conditions such as endocarditis, endophthalmitis, systemic infections, and orthopedic infections [1].

Due to the abundance of *C. acnes* in skin flora and its recent emergence in the setting of wounds and deep-seated infections, a major debate has been sparked about whether or not *C. acnes* is present as a contaminant or a legitimate infection [2]. McLorinan et al. showed through immunofluorescence that the presence of *C. acnes* from surgical wounds in patients who underwent spine surgery was most likely linked to contamination, as it was seen in small aggregates [3]. Carricajo et al. further added to this study by sampling air from operating rooms in addition to demonstrating the presence of *C. acnes*.

*C. acnes* has been associated with both periprosthetic and postoperative joint infections of the shoulder, hip, and knee [4,5,6,7]. Furthermore, *C. acnes* can be seen in cultures from osteomyelitis and postoperative spinal infections [8,9,10,11,12]. However, multiple studies also demonstrate the presence of *C. acnes* in joint injections and disc degeneration [13,14]. Senker et al. found that coagulase-negative Staphylococcus Aureus and *C. acnes* were the two most predominant organisms seen in low-grade infections of intervertebral discs [12]. Similarly, in another prospective study, Coscia et al. also found those two to be the most predominant organisms in intervertebral disc herniation and discogenic pain [13]. Recent research is focusing on *C. acnes* colonization being a potential origin of intervertebral disc degeneration. Schmid et al. demonstrated that *C. acnes* exposure in the intervertebral disc space is seen to upregulate numerous inflammatory markers such as Interleukin (IL)-1β, IL-6, IL-8, and inducible nitric oxide synthase (iNOS), and upregulate the activity of Toll-like receptors (TLR) 2 and 4 [2]. Clinical research studies reported that Modic type 1 changes on MRIs (endplate edema) were concomitant with a positive *C. acnes* culture [15,16,17]. A systematic review by Ganko et al. demonstrated that *C. acnes* was the predominant organism in 59% of disc infections in patients with symptomatic disc degeneration [18]. Nevertheless, the influence of *C. acnes* on the anterior cervical discectomy and fusion (ACDF) remains indeterminate.

Subsidence is a common process after ACDF with an incidence range of 29–54% [19,20,21]. This decreased intervertebral height can result in recurrent nerve root compression, pseudarthrosis, and instability [22,23]. Despite there still being deliberation over whether radiographic evidence of subsidence is clinically significant, several risk factors for subsidence have been recognized. Other risk factors, such as disc degeneration, smoking, low bone density, endplate damage during disc preparation, older age, and cervical malalignment, have each been associated with greater rates of implant subsidence [24,25,26]. However, there is a shortage of literature regarding the effect of *C. acnes* on the incidence of subsidence in ACDF.

This study’s primary objective is to examine the impact of *C. acnes* infection on subsidence after ACDF procedures. The secondary purpose is to assess if *C. acnes* influences postoperative cervical lordosis. Since *C. acnes* has been associated with increased disc degeneration, we hypothesize that positive *C. acnes* cultures from intraoperative disc samples will have an association with increased rates of subsidence.

## 2. Methods

### 2.1. Study Design and Patients

After obtaining Institutional Review Board (IRB) approval (HP-00071677), the study began enrolling consecutive patients from 2017 to 2018 in a prospective cohort study. We adhered to the Strengthening the Report of Observational Studies in Epidemiology (STROBE) guidelines to certify high quality of the study [27]. Inclusion criteria consisted of patients between 18 and 89 years of age with DDD scheduled for elective ACDF. Patients with active malignancy, infection, long-term antibiotic use, and a history of spondylodiscitis or vertebral osteomyelitis were excluded. All of the patients were scheduled for 3- and 6-month follow-ups which included radiographic injury imaging to evaluate the quality of the fusion, cervical lordosis, and the presence of subsidence.

### 2.2. Sample Collection

Every patient received prophylactic antibiotics before the procedure. Patients were positioned supine and standard sterile protocols were observed to avoid skin contamination. Subsequent to the presurgical scrub, the surgical area was sterilized three times with povidone-iodine prior to draping. A standard anterior approach for ACDF was utilized [28]. Once the cervical disc was observed, a sample of disc tissue was obtained with an unused sterile instrument, deposited in a sterile container, and transported to the lab for further microbiological analysis.

### 2.3. Microbiology

Gram-stain and aerobic and anaerobic cultures were ordered for all disc samples. For the aerobic cultures, the samples were homogenized and plated on 5% sheep blood, chocolate, MacConkey, and phenylethyl alcohol agars. For the anaerobic cultures, the samples were homogenized and plated on pre-reduced Brucella blood, Brucella laked blood with kanamycin and vancomycin, Bacteroides bile esculin agar, and thioglycolate broth. The plated agars were incubated at 37 °C in an anaerobic Bio-Bag (Type-A) Multi-plate system and inspected daily for growth. Gram-stain, subculture, and microbial identification using matrix-assisted laser desorption/ionization time-of-flight mass spectrometry (MALDI-TOF MS) were performed to discover any detected colonies [29]. Agar plates were held for 7 days, while the thioglycolate broth was held for 14 days.

### 2.4. Radiographic Measurements

Radiographic measurements were completed independently by 4 separate raters (1 spine attending, 1 spine fellow, 1 orthopedic resident, and 1 medical student) using Surgimap (Version 2.2.15.4, Nemaris Inc, New York, NY, USA). Every rater was blinded to the patients’ culture results. Each rater examined the patient immediately postoperatively, as well as the 3- and 6-month follow-up results, lateral cervical spine radiographs, and measured the cervical lordosis and length of the fused segment. Within 1 month of the initial measurements, each rater also re-measured 20 random patients to confirm intra-rater consistency.

Cervical lordosis was defined as the cobb angle between the inferior end plate of C2 and the superior end plate of C7. The length of the fused segment is the distance between the superior end plate of the superior fused vertebrae to the inferior endplate of the inferior fused vertebrae at the anterior edge of the vertebral body, excluding osteophytes. Fused segment sizes were analyzed for each level of the fusion.

### 2.5. Statistical Analysis

Data were collected and analyzed using Microsoft Excel 365 (Microsoft Office Professional Plus, Microsoft Corporation, Redmond, WA, USA). Continuous variables were established for normality using the Shapiro–Wilk test. Student’s *T* test was employed to evaluate continuous variables with normal distribution and Wilcoxon’s rank-sum test was used as the non-parametric. An a priori alpha was set at 0.05 for significance. Chi-square analysis and Fisher’s exact test were applied to assess the relationships between categorical variables. Post hoc analysis was acheived by multiplied 2 × 2. Fisher’s exact tests for variables with >2 categories that had a statistically significant *p*-value. 

Intra-rater reliability was analyzed for each of the four raters using the Intraclass Correlation Coefficient (ICC). Inter-rater reliability was calculated for cervical lordosis and subsidence measurements using ICC. The mean of the measurements determined by the four raters was used for subsequent analysis. The association between a positive disc culture and cervical lordosis at 3 months and 6 months was evaluated using a mixed effects model, which controlled for the baseline cervical lordosis measure as a fixed effect and incorporated the subjects and raters as a random effect in the model. Likewise, the association between a positive disc culture and discs subsidence at 3 months and 6 months was evaluated using a mixed-effects model, which controlled for baseline measurements and patient characteristics. All analyses were completed using SAS v9.4 statistical software (SAS Institute, Cary, NC, USA).

## 3. Results

### 3.1. Patient Characteristics

A total of 110 patients consented to partake in the study. Of these patients, 14 of them were excluded from the study due to inadequate intraoperative disc sample conditions as characterized by the pathologist (quality of specimen, contamination, etc.). An additional 17 patients were excluded for mixed constructs (ACDF with posterior fusion or standalone and traditional construct). Finally, 13 patients were lost due to a lack of follow-up and did not have imaging. In total, 66 patients met the inclusion criteria and were included in the study. A total of 119 intervertebral disc tissue samples were attained for culture. The cohort had an average age of 53.6 ± 10.1, a BMI of 28.8 ± 5.4 kg/m^2^, and a majority of female patients (n = 36, 54.5%) (Table 1). Neck pain (92.4%) and shoulder pain (89.4%) were the most frequently reported symptoms. The majority of the patients were non-smokers (60.6%) and received two-level fusion (62.1%). Additionally, 40 patients (60.1%) had at minimum one positive disc culture with a total of 79 (66.4%) culture-positive discs (Table 2). Culture-positive patients were younger than culture-negative patients (51.8 vs. 56.4, *p* = 0.042) and less likely to be female (40.0% vs. 76.2%, *p* = 0.003). Furthermore, culture-positive patients were more likely to have received multi-level fusion (82.5% vs. 53.9%, *p* = 0.024). The two cohorts had comparable levels of smoking status, diagnosis, and symptoms (Table 2).

### 3.2. Rater Reliability

Inter-rater reliability was high for cervical lordosis and subsidence measurements between the four raters (ICC = 0.83 and 0.82, respectively) (Table 3). Four raters obtained all measurements on all patients. Additionally, intra-rater reliability was high between the four raters with ICCs greater than 0.84 and an average intra-rater reliability of 0.92.

### 3.3. Cervical Lordosis

Postoperatively, the culture-positive and -negative cohorts had 13.2° and 11.2° of cervical lordosis, respectively. There was no significant difference in the change in cervical lordosis between the two groups at 3 months (2.5°, 95% CI: −1.0°–6.0°, *p* = 0.16) and 6 months (3.0°, 95% CI: −2.4°–8.4°, *p* = 0.27) following surgery.

### 3.4. Subsidence

The culture-positive and -negative cohorts had a mean postoperative fused segment length of 37.0 mm and 36.5 mm, respectively. For the culture-positive cohort, the mean fused segment length decreased to 34.3 mm at 3 months and 35.4 mm at 6 months. In the culture-negative cohort, the mean fused segment length decreased to 33.1 mm at 3 months and 33.6 at 6 months. There was no significant difference in subsidence between culture-positive and -negative cohorts observed at 3 months (1.2 mm, 95% CI: −0.1–2.6 mm, *p* = 0.07) or at 6 months (1.7 mm, 95% CI: −0.4–3.9 mm, *p* = 0.11).

For the most superiorly fused segments, there was no significant difference in subsidence between culture-positive and -negative cohorts observed at 3 months (0.4 mm, 95% CI: −1.6–2.4 mm, *p* = 0.67) and at 6 months (1.1 mm, 95% CI: −1.3–3.5 mm, *p* = 0.36). For the most inferiorly fused segments, there was a significant difference in subsidence observed at 3 months (1.5 mm, 95% CI: 0.2–2.7 mm, *p* = 0.02) but not at 6 months (1.5 mm, 95% CI: −0.2–3.7 mm, *p* = 0.17) between culture-positive and -negative cohorts.

### 3.5. Comparison of Evaluation Metrics

The impact of disc culture on cervical lordosis and subsidence was analyzed using two different metrics: absolute difference and percent difference. Both metrics were further analyzed using a mixed-effects model. The results of the analysis are shown in Table 4. As denoted earlier, all values were normalized to the initial postoperative measurements. The mean difference highlights the difference between culture-positive and culture-negative measurements with culture-negative values predetermined as a reference value of 0. The culture-positive group consisted of cervical lordosis angles that were 2.5 and 3 degrees higher relative to the culture-negative group at 3 and 6 months, respectively. Similarly, the culture-positive group had subsidence measurements that were 1–2 mm higher relative to those of culture-negative groups. The results from the analysis based on percent difference parallel the results from absolute difference.

## 4. Discussion

The pathogenesis of cervical degenerative disc disease (DDD) continues to be inadequately understood. Proposed etiologies include age-related intervertebral disc water loss, reduced endplate permeability, and biomechanical factors [30]. One proposed possible mechanism for DDD is *C. acnes* infection, as *C. acnes* has been demonstrated to be positive in 59% of infectious cases of DDD [18,31]. In the current study, 60% of patients who underwent ACDF had cultures positive for C. *acnes.* Graft subsidence is a usual occurrence in ACDF, which may decrease fusion rates and increase the risk of nearby segment disease [19,20,21]. Bivona et al. reported that 17.7% of patients who received an ACDF had positive intraoperative *C. acnes* cultures [32]. The purpose of this study is to investigate if *C. acnes* colonization in intervertebral discs raises the risk of subsidence following ACDF.

Although *C. acnes* has been linked to postoperative infections, aseptic loosening, and possibly DDD, this study did not establish whether or not *C. acnes* contributes to increased rates of subsidence following ACDF. There was not a significant difference in subsidence at 3 or 6 months postoperatively between the culture-positive and culture-negative cohorts. Both cohorts started to show subsidence at an approximate measurement of 3 mm loss in height by 6 months. Even though the culture-negative cohort had unexpectedly shorter fused segment lengths, the differences in the extent of subsidence were not statistically significant. It is important to distinguish that the two cohorts had different distributions of age, gender, and the number of fused levels. We saw that with a decreased age patients were more likely to have a positive disc culture which is probably due to younger patients having more exposure to *C. acnes* as they have more sebum production [33]. We saw that men were more likely to have positive cultures which is consistent with the current literature [34] as men typically have more sebaceous glands and, resultingly, a higher exposure to *C. acnes*. Additionally, we see that with more levels fused comes an increased risk of a positive culture, which could be due to an exposure issue as multilevel fusions have longer operative times, and the patient’s discs are exposed to the skin’s flora for a longer period of time. However, when these elements were controlled for in the mixed-effects model, the *C. acnes*-positive cohort had an increased, but not statistically significant, degree of subsidence.

Past studies have also mentioned that subsidence may play a crucial role in preserving sagittal balance, particularly in maintaining the natural cervical curvature following ACDF [35,36]. In this study, there was no significant difference in the loss of lordosis between culture-positive and culture-negative groups up to 6 months postoperatively. Conversely, larger studies with longer follow-up periods may be required to ascertain the significant effect of subsidence on cervical lordosis.

To the authors’ knowledge, this is the first prospective study of its kind to investigate subsidence rates following ACDF via the collection of disc samples cultured for *C. acnes*. Future studies are necessary to validate these findings and compare the differences in subsidence with clinical outcomes, such as reoperation rates and patient-reported outcomes. If future studies discover an association between *C. acnes* and subsidence after ACDF, clinical management may be altered to include prophylactic preoperative or postoperative antibiotics. As *C. acnes* research further develops, an organism that was once thought to be a contaminant in cultures may become a key to fully comprehending DDD and postoperative subsidence.

There are numerous limitations to this study. Though measurements of subsidence emphasized high intra- and inter-rater reliability, these small measurements demonstrated no statistical significance. Although our sample size was not sufficient for binary variables, we were sufficiently powered for continuous variables after post hoc power analysis. Future studies can focus on enrolling a larger sample size, allowing for the necessary power to focus on subsidence as a binary variable instead of a continuous variable. Future studies can also focus on longer follow-up data since this paper focuses on short-term follow-up outcomes. Lastly, the association between radiographic measurements of subsidence and clinical significance continues to be a topic of debate. Nevertheless, this prospective study provides vital early evidence and an initial framework for future studies of the effect of *C. acnes* on subsidence following ACDF.

## 5. Conclusions

Intervertebral discs with a positive *C. acnes* culture were not associated with greater levels of subsidence at 3 and 6 months after ACDF for DDD. Future research is warranted to corroborate these results and to further explore the clinical significance of *C. acnes* infection.

## Figures and Tables

**Table 1:** Characteristics of Patients Included in the Study.

Demographics	
Number of Patients	66
Number of Discs	119
Age, (Mean ± SD)	53.6 ± 10.1
BMI, (Mean ± SD)	28.8 ± 5.4
Sex (Male), N (%)	30 (45.5)
Previous C-Spine Surgery, N (%)	7 (10.6)
Diagnosis, N (%)	
Cervical Myelopathy	12 (18.2)
Cervical Radiculopathy	26 (39.4)
Cervical Stenosis	10 (15.2)
Disc Herniation	11 (16.7)
Cervical Spondylosis	7 (10.6)
Symptoms, N (%)	
Neck Pain	61 (92.4)
Arm/Shoulder Pain	59 (89.4)
Smoking Status, N (%)	
Non-Smoker	40 (60.6)
Current Smoker	14 (21.2)
Former Smoker	12 (18.2)
Segments, N (%) †	
C3/C4	9 (7.5)
C4/C5	28 (23.5)
C5/C6	51 (42.8)
C6/C7	31 (26.0)
C7/T1	1 (0.8)
Levels Fused, N (%)	
One	19 (28.8)
Two	41 (62.1)
Three	6 (9.1)

† Total number of discs (119) used for percent calculation. SD—standard deviation.

**Table 2:** Patient Characteristics with Positive and Negative Disc Cultures.

Characteristics	Negative Disc Culture	Positive Disc Culture	*p*-Value
Number of Patients	26	40	
Number of Discs	40	79	
Age	56.4 ± 6.2	51.8 ± 11.6	**0.04**
BMI	28.4 ± 5.5	29.0 ± 5.3	0.66
Sex (Male)	6 (23.8)	24 (60.0)	**<0.01**
Previous C-Spine Surgery	3 (11.5)	4 (10.0)	1.00
Diagnosis, N (%)			0.93
Cervical Myelopathy	5 (19.2)	7 (17.5)	
Cervical Radiculopathy	11 (42.3)	15 (37.5)	
Cervical Stenosis	3 (11.5)	7 (17.5)	
Disc Herniation	5 (19.2)	6 (15.0)	
Cervical Spondylosis	2 (7.7)	5 (12.5)	
Symptoms, N (%)			
Neck Pain	25 (96.2)	36 (90.0)	0.34
Arm/Shoulder Pain	22 (84.6)	37 (92.5)	0.28
Smoking Status, N (%)			0.61
Non-Smoker	14 (53.8)	26 (65.0)	
Current Smoker	7 (26.9)	7 (17.5)	
Former Smoker	5 (19.2)	7 (17.5)	
Levels Fused, N (%)			**0.01**
One	12 (46.5)	7 (17.5)	**0.02**
Two	14 (53.9)	27 (67.5)	0.31
Three	0 (0)	6 (15.0)	0.07
Follow Up-Weeks (Mean ± SD)			
Immediate Post op	0.2 ± 0.7	0.2 ± 0.5	0.71
T1 (weeks)	17.1 ± 6.4	16.9 ± 5.5	0.91
T2 (weeks)	42.4 ± 15.7	43.3 ± 13.2	0.86

**Table 3:** Intra- and Inter-Rater Reliability for Cervical Lordosis and Fused Segment Length.

Measurements	ICC	95% CI
Intra-Rater Reliability †		
1	0.93	0.84–0.97
2	0.91	0.79–0.96
3	0.99	0.96–0.99
4	0.84	0.64–0.93
Inter-Rater Reliability		
Cervical Lordosis	0.83	0.76–0.88
Fused Segments	0.82	0.77–0.86

† Intra-rater reliability was calculated by repeat measurement of 20 samples by each rater. ICC—Intraclass Correlation Coefficient; CI—confidence interval.

**Table 4:** Impact of Disc Culture on Change in Cervical Lordosis and Subsidence.

	Follow Up	Mean Diff	95% CI	*p*-Value
Absolute Difference †				
Cervical Lordosis	3 Months	2.5	−1.0–6.0	0.16
	6 Months	3	−2.4–8.4	0.27
Subsidence: Combined	3 Months	1.2	−0.1–2.6	0.07
	6 Months	1.7	−0.4–3.9	0.11
Subsidence: Superior Level	3 Months	0.4	−1.6–2.4	0.67
	6 Months	1.1	−1.3–3.5	0.36
Subsidence: Inferior Level	3 Months	1.5	0.2–2.7	**0.02**
	6 Months	1.5	−0.7–3.7	0.17
Percent Difference *				
Cervical Lordosis	3 Months	0.9%	0.3–2.2	0.15
	6 Months	0%	−1.2–1.2	0.99
Subsidence: Combined	3 Months	2.6%	−0.9–6.0	0.14
	6 Months	1.9%	−2.1–5.9	0.33
Subsidence: Superior Level	3 Months	1.1%	−6.8–4.6	0.70
	6 Months	2.0%	−4.3–8.4	0.52
Subsidence: Inferior Level	3 Months	0.4%	0.2–7.0	**0.04**
	6 Months	1.4%	−3.4–6.2	0.55

† Mean difference in positive and negative disc cultures at 3-month and 6-month follow-up. Negative disc cultures were used as a reference point for the mixed model. * Percent difference calculated as follow up/pre-operative measurements. CI—confidence interval.

## Data Availability

Data are available upon request from authors.
